# COVID-19 Vaccine in Pregnant and Lactating Women: A Review of Existing Evidence and Practice Guidelines

**DOI:** 10.3390/idr13030064

**Published:** 2021-07-31

**Authors:** Ishan Garg, Rahul Shekhar, Abu B. Sheikh, Suman Pal

**Affiliations:** 1 Mayo Clinic College of Medicine, Mayo Clinic, Rochester, MN 55902, USA; ishangargmd@gmail.com; 2 Department of Internal Medicine, University of New Mexico Health Sciences Center, Albuquerque, NM 87106, USA; rshekhar@salud.unm.edu (R.S.); absheikh@salud.unm.edu (A.B.S.)

**Keywords:** Coronavirus 2019, COVID-19, vaccine, COVID-19 vaccines, pregnancy, lactation, breast feeding, fertility

## Abstract

Coronavirus 2019 (COVID-19) has created a global pandemic that is devastating human lives, public healthcare systems, and global economies. Multiple effective and safe COVID-19 vaccines have been developed at an unprecedented speed due to the efforts of the scientific community, and collaboration between the federal government and pharmaceutical companies. However, the continued exclusion of pregnant and lactating women from the COVID anti-viral and vaccine trials has created the paradox of a lack of empirical evidence in a high-risk population. Based on the experience of similar prior vaccines, animal developmental and reproductive toxicology studies, and preliminary findings from human studies, various healthcare professional advisory committees (Advisory Committee on Immunization Practices, American College of Obstetricians and Gynecologists, Society for Maternal-Fetal Medicine, and Academy of Breastfeeding Medicine) have issued guidance supporting COVID-19 vaccination in pregnant and lactating women. In this article, we summarize the available data on the efficacy and safety profile of COVID-19 vaccination in pregnant and lactating women, review the challenges of vaccine hesitancy, and include recommendations for healthcare providers.

## 1. Introduction 

The coronavirus disease 2019 (COVID-19) pandemic has created a global health crisis of unprecedented proportions. It is caused by the severe acute respiratory syndrome coronavirus 2 (SARS-CoV-2). More than 173 million cases and 3.7 million deaths have been attributed to COVID-19 globally [1]. Along with this devastating loss of human life, the COVID-19 pandemic has also put an extraordinary strain on public healthcare systems and global economies [2]. Vaccination and public health measures, including wearing facemasks, social distancing, and personal hygiene, remain the most promising means of controlling this global pandemic. With this hope, multiple COVID-19 vaccines are being developed, approved, and manufactured for global use while still upholding rigorous regulatory processes [3,4,5]. 

It is important to note that pregnant and lactating women were excluded from clinical trials of COVID-19 vaccines, despite being at increased risk of developing severe illness from COVID-19 compared with non-pregnant females [6,7,8,9,10,11,12,13,14,15]. Pregnant females with COVID-19 are also at increased risk of hospitalization, intensive-care unit (ICU) admission, invasive ventilation support, and preterm birth compared with pregnant females without COVID-19 [14,15,16]. Several studies have also shown a small, 2% to 3% risk of vertical transmission and the presence of viral RNA in breast milk of mothers infected with COVID-19 [17,18,19,20,21,22,23,24,25,26].

Millions of women have become pregnant, given birth, and initiated breastfeeding since the start of the COVID-19 pandemic. This has created an ethical and clinical conundrum on protecting our vulnerable pregnant and lactating population while lacking empirical evidence. Based on limited data, advisory committees from the Centers for Disease Control and Prevention (CDC) and Advisory Committee on Immunization Practices (ACIP), American College of Obstetricians and Gynecologists (ACOG), and the American Academy of Pediatrics (AAP) have issued guidance indicating that COVID-19 vaccines should not be withheld from pregnant persons [6,27,28].

The purpose of this article is to provide an overview of the efficacy and safety of COVID-19 vaccination in pregnant and lactating women, with a review of limited data and theoretical considerations and review-recommended guidelines for clinical use.

## 2. COVID-19 Vaccines

Scientific ingenuity combined with federal government support has led to the development of several vaccine candidates for COVID-19 at a breakneck speed. The US Department of Health and Human Services announced the framework for Operation Warp Speed, a partnership approach between the U.S. Government and the pharmaceutical industry, on 15 May 2020, to accelerate the development of safe, effective COVID-19 vaccines. At present, there are 93 vaccines under clinical trials on humans and at least 77 preclinical vaccines under investigation in animals [29].

Six leading vaccine candidates have received some form of federal government support through Operation Warp Speed. These can be divided into three different types based on their mechanism of action: messenger RNA (mRNA) (BNT162b2 Pfizer BioNTech (Pfizer, Inc., Philadelphia, PA, USA) and mRNA-1273 Moderna vaccines (ModernaTX, Inc., Cambridge, MA, USA)), viral vector (AstraZeneca, Janssen Ad26.COV2.S) (Janssen Biotech, Inc, Johnson & Johnson, New Brunswick, NJ, USA), and recombinant antigen proteins manufactured in a baculovirus (DNA virus that infects insect cells) system (Novavax (Novavax, Inc., Gaithersburg, MD, USA) and GSK-Sanofi (Sanofi Inc., Bridgewater, NJ, USA; GlaxoSmithKline Inc., Philadelphia, PA, USA). 

Moderna was the first one to be granted Emergency Use Authorization (EUA) by the FDA. EUA is an authority provided to the FDA to allow unapproved medical products to be used in a public health emergency to diagnose, treat, or prevent serious or life-threatening conditions when there are no adequate, approved, and available alternatives [30]. However, it is worth noting that the FDA implemented a more rigorous review process to maintain higher standards for COVID-19 vaccine, given that they would be used on a large population [31]. Since then, various other vaccines have been approved. The COVID-19 vaccines that have been approved by WHO under the emergency use listing (EUL) include Pfizer/BioNTech, Moderna, Janssen, AstraZeneca (produced by SK Bio and Serum Institute of India—Covishield), Sinopharm (China National Pharmaceutical Group, Beijing, China), and Sinovac-CoronaVac (Sinovac Biotech Ltd., Beijing, China) vaccines.

### 2.1. mRNA 

The Pfizer-BioNTech and Moderna vaccines are lipid nanoparticle mRNA vaccines. The vaccine mRNA encodes for the SARS-CoV-2 spike protein, which attaches the virus to angiotensin-converting enzyme 2 (ACE2) to initiate the infection process. The lipid nanoparticles facilitate entry into the cell at the site of injection. The mRNA is then transcribed in the host cell, producing the spike protein, which is subsequently presented on the cell surface to B and T cells, resulting in immune response [32,33,34,35,36,37].

Both of these FDA-approved mRNA vaccines have shown robust immune response and protection against severe COVID-19 [38,39]. The reported efficacy of Pfizer-BioNTech and Moderna vaccines is 95% and 94%, respectively, in preventing COVID-19 in participants of phase III clinical trials (16 years and older for Pfizer/BioNtech vaccine and 18 years or older for Moderna vaccine) [38,39,40]. Common side effects included injection site reactions (84.1% for the Pfizer/BioNTech vaccine, 91.6% for the Moderna vaccine), fatigue (62.9%, 68.5%), headache (55.1%, 63.0%), muscle pain (38.3%, 59.6%), chills (31.9%, 43.4%), joint pain (23.6%, 44.8%), and fever (14.2%, 14.8%), all of which generally resolve within a day or two. On rare occasions, mRNA vaccines can trigger myocarditis, anaphylaxis, or Bell’s palsy. Therefore, the CDC recommends monitoring vaccine recipients 15 min after their COVID-19 shot or for 30 min if they have a history of severe allergies [41,42,43]. Pfizer-BioNTech vaccine is currently approved for individuals aged 12 years or older, while the Moderna vaccine is approved for 18 years or older. Both vaccines require two doses, 21 (Pfizer-BioNTech, Brooklyn, NY, USA) to 28 days (Moderna, Cambridge, MA, USA) apart [44].

### 2.2. Viral Vector

These vaccines, for example, Oxford-AstraZeneca and Johnson & Johnson Janssen Ad26.COV2.S vaccines use viral vectors for the delivery of spike protein RNA into the host cell. The viral vector is made from a modified version of a harmless (modified non-replicating) adenovirus. The final vaccine product contains the spike protein found in SARS-CoV-2 and elicits an immune response via a similar mechanism to mRNA-based vaccines.

Oxford/AstraZeneca vaccine requires two doses, while the Janssen vaccine is authorized for use as a single dose vaccine. At present, only the Janssen vaccine has EUA for use in the U.S for individuals aged 18 years or older. [45].

Common side effects of the Janssen vaccine include injection site pain [48.6%], headache [38.9%], fatigue [38.2%], and myalgia [33.2%], all of which generally resolve within a day or two. The severity of these symptoms is noted to be considerably less than the mRNA-based vaccines. On rare occasions, Janssen vaccine can cause radiculitis and Guillain-Barre syndrome. Other rare, albeit serious, potentially life-threatening cases of thrombosis with thrombocytopenia syndrome (TTS) have been noted following the Janssen COVID-19 vaccine [46]. Interestingly, all the 15 reported cases (after 7.98 million vaccine doses) have been reported in females. Pregnancy, hormone therapy, or birth control pills are pre-existing risk factors for thrombosis [47]. Therefore, it is advisable to counsel women younger than 50 years old about the increased risk of thrombosis with the Janssen vaccine [48]. The salient features of the Moderna, Pfizer- BioNTech, and Janssen vaccines are summarized in Table 1.

### 2.3. Recombinant Antigen Proteins

These vaccines, for example, Novavax and GSK-Sanofi, use protein subunits combined, mixed with an adjuvant (an adjuvant is an ingredient used to strengthen the immune response). Unlike other vaccines, these vaccines contain the spike protein itself and, when injected into the body, illicit an immune response like other vaccines. These vaccines are still in clinical trial phases and have not been approved for use. The reported efficacy of NOVAX is 96.4% in reducing mild and moderate disease and 100% against severe disease from the COVID-19 [49].

### 2.4. Inactivated COVID-19 Virus

These vaccines, such as WIV04 and HB02 (Sinopharm, Beijing, China), Coronavac (Sinovac, Beijing, China), use inactivated COVID-19 virus to trigger an immunologic response in the host. Both Sinopharm and Sinovac use aluminum hydroxide as adjuvant. These vaccines do not contain the live virus and therefore cannot cause the disease. The reported efficacy of Sinopharm and Sinovac is 73% (40,000 participants) and 83.5% (10,000 participants; preventing COVID-19 in those without prior infection) from the phase III trials, respectively. Both vaccines require two doses. Common side effects included injection site reactions, fatigue, headache, muscle pain, chills, joint pain, and fever, all of which generally resolve within a day or two. These vaccines are available in China and some other countries, including the United Arab Emirates, Brazil, Chile, Indonesia, Mexico, Turkey, and Hungary. At present, these vaccines are not approved for use in the United States.

## 3. Clinical Trials of COVID-19 Vaccine in Pregnant and Lactating Women

Pregnant and lactating women are usually not included in the vaccine research, primarily because of safety and liability concerns, not only for the mother but also the baby [50,51,52,53,54]. However, systemic exclusion of this vulnerable population from clinical trials, including from SARS-CoV-2 vaccine trials, also means limited available data on vaccine safety and efficacy in pregnant and lactating women. 

At present, there are over 300 clinical trials exploring new medications and vaccines for COVID-19; however, pregnant women are excluded from all these trials [55]. However, Pfizer (23 pregnancies) and Moderna (12 pregnancies) reported that a few participants inadvertently became pregnant during the trials.

Several efforts have been made over the years to address the exclusion of pregnant women from clinical trials of new medications and vaccines, including [56,57], for example, the National Institute of Allergy and Infectious Diseases has developed guidelines for protocol design and safety assessment for clinical trials conducted in pregnant persons. In 2018, the FDA provided a framework for the inclusion of pregnant persons in clinical trials [58]. The Task Force on Research Specific to Pregnant Women and Lactating Women recommends, “inclusion of pregnant women and lactating women in clinical trials, unless there are compelling scientific reasons for their exclusion [59].” Despite these efforts, very little actual progress has been made as this population continues to be excluded from clinical trials.

In recognition of the importance of the inclusion of pregnant women in COVID-19 vaccine clinical trials, the FDA recommended conducting developmental and reproductive toxicology studies (DART) before enrolling pregnant people or persons who are not actively avoiding pregnancy in clinical trials [60]. The developmental and reproductive toxicology studies are designed to study the effect of new medication or vaccines on the full spectrum of reproduction in animals. mRNA-based Moderna vaccine submitted these results to the FDA on December 4, 2020 [61]. Pfizer has recently announced a global Phase 2/3 trial to evaluate the safety, tolerability, and immunogenicity of the COVID-19 vaccine in pregnant women [62]. The trial is a randomized, placebo-controlled, observer-blind study and will include 4000 healthy women vaccinated between 24 and 34 weeks of gestation [63]. Modera has announced a prospective observational study to assess obstetric, neonatal, and infant outcomes and has created a registry. Johnson & Johnson subsidiary Janssen is also planning a phase 2 placebo-controlled trial including 824 pregnant females [64].

CDC has also created a voluntary smartphone-based app called “v-safe,” which includes pregnant women, to register voluntary reporting of adverse events following COVID-19 vaccination. Data collected so far include over 50,000 pregnant women and show no serious vaccine-related adverse events [65]. The United Kingdom has also created a similar registry for its citizens, showing similar results with no safety concerns related to COVID-19 vaccination [66]. The University of Washington has also established a similar registry involving individuals who are pregnant, postpartum, lactating, or contemplating pregnancy in the next 1 or 2 years. Harvard School of Public Health is also working on developing a registry to evaluate obstetric, neonatal, and infant outcomes after COVID-19 vaccination during pregnancy. Modi et al. suggest that regulators should mandate the development of “pregnancy-investigation plans” to describe the development pathway for vaccines and medicines that will be used in pregnancy in an approach similar to the European Medicines Agency’s “pediatric investigation plans” for medicines used in children [67,68].

A combination of (i) strong advocacy groups providing regulatory framework and guidelines for inclusion of pregnant and lactating women in COVID-19 vaccine clinical trials, and (ii) leveraging data collected DART studies, accidental pregnancies during clinical trials, and various safety profile registries can help bridge the gap between evidence-based recommendations and expert opinion on COVID-19 vaccination in pregnant and lactation women [69,70,71].

## 4. Impact of COVID-19 Vaccine in Pregnant Women

Despite potentially devastating consequences of COVID-19 infection in pregnant women and the availability of safe and efficacious (in non-pregnant populations) COVID-19 vaccination, there is limited published data describing the safety or efficacy of any COVID-19 vaccine in human pregnancy. Approval of vaccine use intended for pregnant or breastfeeding women rely on a critical review of observational studies, clinical case reports, registries, and clinical trials to ascertain if the vaccine is: (i) safe—no adverse pregnancy outcomes or potential harm to mother and/or fetus, (ii) efficacious—reduce morbidity in the pregnant woman and/or fetus [72,73,74,75,76,77,78,79,80,81]. These reviews are conducted through collaborative efforts between the World Health Organization (WHO), CDC, National Institutes of Health (NIH), ACIP, Task Force on Research Specific to Pregnant Women and Lactating Women, and Global Advisory Committee on Vaccine Safety, among others.

Data from Pfizer/BioNTech, Moderna, and Janssen vaccines’ animal DART studies have found no safety concerns with no adverse effect on female reproduction, fertility, fetal or embryonal, or postnatal development, miscarriage [6,11,38,39,82,83,84,85]. Preliminary human studies on mRNA-based vaccines, including the Zika virus, influenza virus, and rabies virus, have shown good safety and immunogenicity profile during pregnancy [86,87,88,89,90,91,92].

In a prospective cohort study including 131 reproductive-age vaccine recipients (84 pregnant, 31 lactating, and 16 non-pregnant women), Gray et al. reported that mRNA-based COVID-19 vaccines generated robust humoral immunity in pregnant and lactating women, with immunogenicity and reactogenicity similar to that observed in non-pregnant women. They also reported the transfer of protective immunoglobulins to neonates via placenta and breastmilk [93].

Shimabukuro et al. analyzed data on the safety of mRNA COVID-19 vaccines in pregnant women from the safety surveillance registries, including “v-safe” and Vaccine Adverse Event Reporting System (VAERS). Their analysis included 35,691 pregnant v-safe participants. They reported injection-site pain, fatigue, headache, and myalgia as the most frequent local and systemic reactions after vaccination, which were more frequent after the second dose. The participant-measured temperature at or above 38 °C was reported by less than 1% of the participants on day one after dose one and by 8.0% after dose 2 for both vaccines. They commented that these patterns, including higher reporting of reactogenicity after dose 2, were similar to patterns observed among non-pregnant women. Pregnant women did not report having severe reactions more frequently than non-pregnant women, except for nausea and vomiting, which were reported slightly more frequently only after dose 2.

Small numbers of pregnant people were inadvertently enrolled during vaccine clinical trials of Pfizer/BioNTech (23, including 11 in the vaccine arm), Moderna (13, including six in the vaccine arm), and Janssen vaccine (8, including 4 in the vaccine arm) [61,83,94,95]. They only reported unsolicited data on adverse events related to pregnancy, including miscarriage. In Pfizer/BioNTech (1/12; 8%) and Moderna vaccine trials (1/7; 14%) reported miscarriage only in the placebo group. In the Janssen vaccine trial, reported adverse events during pregnancy included spontaneous abortion (1 vaccine, 0 placebo), incomplete abortion (0 vaccine, 1 placebo), elective abortion (0 vaccine, 2 placebo), and ectopic pregnancy (1 vaccine, 0 placebo). The results are summarized in Table 2.

## 5. COVID-19 Vaccine Hesitancy

Despite tremendous progress in vaccine development and administration, the current acceptance level of the COVID-19 vaccine remains inadequate to meet the requirements for developing herd immunity. The herd immunity threshold depends on the basic reproduction number of the disease. Assuming a basic reproductive number of 4, the community immunity level needs to reach at least 75% to stop the COVID-19 pandemic [96]. WHO has categorized vaccine hesitancy as one of the top ten threats to global health, even prior to the COVID-19 pandemic [97,98]. Therefore, it is important to understand and address the reason for vaccine hesitancy.

Skjefte et al. collected data from 17,871 respondents from 16 countries through an online survey between October 28 and November 18, 2020, assuming a hypothetical 90% COVID-19 vaccine efficacy. Only 52.0% of pregnant women (*n* = 2747/5282) and 73.4% of non-pregnant women (*n* = 9214/12,562) indicated an intention to receive the vaccine [98]. They noticed the top three reasons given by pregnant women to decline COVID-19 vaccination during pregnancy, even if the vaccine were safe and free, were: (i) that they did not want to expose their developing baby to any possible harmful side effects (65.9%), (ii) were concerned that approval of the vaccine would be rushed for political reasons (44.9%), and (iii) would like to see more safety and effectiveness data among pregnant women (48.8%).

Vaccine hesitancy is a complex, multifaceted problem with significant variability based on region, race, ethnicity, pregnancy, education level, employment status, social and geopolitical influence [99]. Various steps, including rebuilding public vaccine confidence by uniform, consistent, transparent, and effective communication from policymakers, media, and health care providers, and effective community engagement are important to improve vaccine acceptance [98,99,100].

## 6. Impact of COVID-19 Vaccine on Fetuses and Breastfeeding Infants

Vaccination during pregnancy is known to provide protection to mother and infant from infectious agents including, e.g., influenza and pertussis. Various expert panels suggest that COVID-19 vaccine based on mRNA nanoparticle and adenovirus vector do not possess any significant risk to the fetus or breastfeeding infant [7,10,11,12,101,102,103], although there is no direct data on whether intact vaccine particles cross the placenta and enter fetal cells. However, studies done on other lipid nanoparticle-based vaccines suggest that they cannot cross the placenta [86,104,105]. Another small study including six lactating women showed no evidence of vaccine mRNA in breast milk samples collected within two days of vaccination [106].

From the analysis of the safety surveillance registries, including “v-safe” and Vaccine Adverse Event Reporting System (VAERS), Shimabukuro et al. reported pregnancy loss occurred in 13.9% of participants that had a completed pregnancy (i.e., live-born infant, spontaneous abortion, induced abortion, or stillbirth). They also note adverse neonatal outcomes of preterm birth (9.4%) and small size for gestational age (3.2%). No neonatal deaths were reported. Among 221 pregnancy-related adverse events reported to the VAERS, the most frequently reported event was spontaneous abortion (46 cases). Similar incidences of these pregnancy outcomes have been reported in studies involving pregnant women that were conducted before the COVID-19 pandemic [107,108,109,110,111,112].

Many experts have also suggested that vaccine-stimulated immunoglobulin A may pass through breast milk and provides additional protection to the infant against COVID-19 [86,104,105,106,113,114]. One study showed the presence of vaccine-derived IgA antibodies in breastmilk three to four weeks post-vaccination with the mRNA COVID-19 vaccines (*n* = 23) [106]. They also noted that IgA antibody titers in breastmilk were similar between participants receiving COVID-19 vaccination and COVID-19 infection. Another study, including 20 mother-infant pairs, demonstrated efficient transplacental transfer of anti-COVID-19 spike antibodies after antenatal vaccination with the Pfizer/BioNTech vaccine [113].

## 7. Impact of COVID-19 Vaccine on Fertility

For people in the reproductive age group or planning pregnancy, the effect of the COVID-19 vaccine is an important consideration. Fortunately, based on multiple reports, no evidence of an adverse effect on the fertility of women or men has been found [115,116,117,118,119,120].

It is interesting to note that COVID-19, in theory, may affect female fertility by damaging endometrial epithelial cells (affecting early embryo implantation), ovarian tissue, and granulosa cells (decreasing ovarian function and oocyte quality) by the release of pro-inflammatory cytokines [117,118,119,120]. However, no such clinical case has yet been reported.

According to the guidance issued by the British Fertility Society, there is “absolutely no evidence” that COVID-19 vaccines can affect the fertility of women or men [121]. They also stated that people undergoing fertility treatment (in vitro fertilization), frozen embryo transfer, egg freezing, ovulation induction, intrauterine insemination, donating eggs or sperms could receive the COVID-19 vaccine. However, they recommended separating the date of vaccination from treatment procedure by a few days to correctly attribute symptoms such as fever to vaccine or treatment procedure.

Orvieto et al. studied 36 couples undergoing IVF treatment 7–85 days after receiving the mRNA COVID-19 vaccine. They reported no in-between cycle differences in ovarian stimulation and embryological variables before and after receiving the mRNA COVID-19 vaccine. They also noted that the mRNA COVID-19 vaccine did not affect patients’ performance or ovarian reserve in their immediate subsequent IVF cycle.

## 8. Recommendations for Health Care Providers

The paucity of data describing COVID-19 vaccine safety, immunogenicity, and efficacy in pregnant and breastfeeding women has made the decision for vaccination a challenging one for many. However, multiple professional societies have offered recommendations to assist physicians and pregnant and breastfeeding women in making such decisions [104].

Health care providers should use a framework to support shared decision-making, providing evidence-based information and allowing the individual to weigh the risk and benefits of vaccination to make a well-informed decision. Health care providers should also try to identify the challenges during inpatient counseling and provide evidence-based and practical guidance addressing those challenges. For example, for vaccine hesitancy, physicians should ask patients to express their hesitation and reasons for it and respectfully address them.

The CDC’s ACIP, ACOG, Society for Maternal-Fetal Medicine (SMFM), and Academy of Breastfeeding Medicine (ABM) recommend that all pregnant and breastfeeding people may choose to be vaccinated and that a discussion with their physician might assist them in making an informed decision [85,101,102,104,108]. Guidelines by various professional committees are summarized in Table 3.

### Special Considerations

The CDC recommends that individuals with a severe and immediate allergic reaction to a previous dose of an mRNA COVID-19 vaccine, its components, or to polysorbate should not receive mRNA COVID-19 vaccination unless they have been evaluated by an allergist-immunologist, and it has been established that the individual can safely receive the vaccine.

Rare, albeit potentially life-threatening, cases of thrombosis have been reported after the administration of the Janssen vaccine. Pregnancy is a risk factor for thrombosis; therefore, pregnant women should be made aware of the increased risk for thrombosis with the Janssen vaccine. However, based on available data (15 cases after 7.98 million doses), the CDC recommends that the vaccine can still be given during pregnancy, as it continues to collect more clinical data [48,122,123].

## 9. Limitations

There are limitations to this review. Our review only included data from FDA and WHO-approved COVID-19 vaccines. Several other potential COVID-19 vaccines remain under investigation. Furthermore, due to heterogeneity in population, vaccine research guidelines, and regulations among various countries, the head-to-head comparison of various other COVID-19 vaccines remains beyond the scope of this article. The literature search for this review included English-language articles only.

## 10. Conclusions

COVID-19 vaccination is the most promising means of controlling the spread of the COVID-19 global pandemic. It is important to safeguard our vulnerable population of pregnant and lactating women while also prioritizing their involvement in clinical trials for vaccine and anti-viral therapies and vaccine administration. Health care providers need to keep up to date with the new information to provide evidence-based information and effective counseling to pregnant and lactating women.

## Figures and Tables

**Table 1:** COVID vaccines granted an Emergency Use Authorization (EUA) by Federal Drug Administration (FDA) and/or Emergency use listing (EUL) by WHO.

	Pfizer/BioNTech	Moderna	Janssen	Oxford/AstraZeneca	Sinopharm	SinoVac
Target Population	Age 12 and older	Age 18 and older				
Vaccine Administration	2 doses, 21 days apart	2 doses, 28 days apart	1 dose	2 doses, 8 to 12 weeks apart	2 doses, 21 to 28 days apart	
Efficacy	95%	94.1%	72% overall and 86% against severe disease	63.1%	73%	83.5%
Side effects	Most common—injection site pain, fatigue, headache, muscle pain, joint pain, & fever					
	More common after the second dose		-	More common after the second dose		
	Rare—Severe allergic reaction Bell’s palsy, myocarditis, & pericarditis		Rare—TTS, post-vaccination syndrome, radiculitis, GBS		N/A	N/A
Safety in Pregnant/Lactating women	Animal Studies—No safety concerns				Animal Studies -N/A	
	Human Studies—N/A					

N/A—Not Available, GBS—Guillain-Barre syndrome, TTS—thrombosis with thrombocytopenia syndrome.

**Table 2:** Accidental pregnancies reported in COVID-19 vaccine trials.

Vaccine Type	Control/Placebo Group			Vaccine Group		
	Participants	Pregnancies	Miscarriage (rate)	Participants	Pregnancies	Miscarriage (Rate)
Pfizer/BioNTech	18,846	12	1 (8%)	18,860	11	0
Moderna	15,170	7	1 (14%)	15,181	6	0
Janssen	21,888	4	1 (25%)	21,895	4	1 (25%) + 1 (25%) *
AstraZeneca	5,829	9	3 (33%)	5,807	12	2 (17%)

* = ectopic pregnancy.

**Table 3:** Guidelines by various professional committees for COVID vaccination in pregnant and lactating women.

Pregnant and Considering Pregnancy	Breastfeeding
Advisory Committee on Immunization Practices (ACIP)	
People who are pregnant and part of a group recommended to receive COVID-19 vaccination, such as health care personnel, may choose to be vaccinated	No data on the safety of COVID-19 vaccines in lactating mothers or on the effects of mRNA vaccines on the breastfed infant
b. A conversation with a clinician may be helpful but is not required prior to vaccination	b. Individuals who meet criteria for vaccination based on ACIP-recommended priority group, may choose to be vaccinated
c. People who are trying to become pregnant do not need to avoid pregnancy after receiving an mRNA COVID-19 vaccine	
American College of Obstetricians and Gynecologists (ACOG)	
COVID-19 vaccines should not be withheld from pregnant individuals who meet criteria for vaccination based on ACIP-recommended priority groups	COVID-19 vaccines should not be withheld from breastfeeding individuals who meet criteria for vaccination based on ACIP-recommended priority groups
b. While a conversation with a clinician may be helpful, it should not be required prior to vaccination	b. Theoretical risks of vaccinating lactating people do not outweigh the potential benefits of the vaccine
c. Pregnancy testing is not a requirement prior to receiving the COVID-19 vaccine	
Society for Maternal-Fetal Medicine (SMFM)	
Discuss potential benefits and unknown risks with their clinicians regarding receipt of vaccine	Vaccination recommended for lactating women
b. Individual decision-making needs to balance theoretical risks with risks associated with delayed vaccination and possible maternal SARS-CoV-2 infection	b. Counseling should balance lack of data on vaccine safety and a person’s individual risk for infection and severe disease
c. Counseling should also include the theoretical risk of harm to the fetus	c. Theoretical risks of vaccinating lactating people do not outweigh the potential benefits of the vaccine
Academy of Breastfeeding Medicine (ABM)	
-	Does not recommend cessation of breastfeeding for individuals who are vaccinated against COVID-19
-	b. Discuss potential benefits and unknown risks with their clinicians regarding receipt of vaccine
-	c. While there is a little plausible risk for the child, there is a biologically plausible benefit
Food and Drug Administration (FDA)	
Insufficient available data on COVID-19 vaccine-associated risks in pregnancy	Insufficient available data on the effect of COVID-19 vaccine- on the breastfed infant or on milk production/excretion

## Data Availability

Not applicable.
